# Matrix metalloproteinase-9 genetic variation and primary angle closure glaucoma in a Caucasian population

**Published:** 2011-05-27

**Authors:** Mona S. Awadalla, Kathryn P. Burdon, Abraham Kuot, Alex W. Hewitt, Jamie E. Craig

**Affiliations:** 1 Ophthalmology Department, Flinders University, Adelaide, South Australia, Australia; 2 Centre for Eye Research Australia, University of Melbourne, Royal Victorian Eye and Ear Hospital, Melbourne, Australia

## Abstract

**Purpose:**

To investigate the association between genetic variation at the matrix metalloproteinase-9 (*MMP9*) locus and primary angle closure glaucoma (PACG) in an Australian Caucasian population.

**Methods:**

A total of 107 Australian patients with PACG and 288 age and sex-matched controls were included in the current study. Tag single nucleotide polymorphisms (SNPs) were selected and genotyped to cover the majority of common variation within *MMP9*. Allele, genotype and haplotype association analyses were conducted using PLINK.

**Results:**

Two SNPs from *MMP9*, rs3918249 and rs17576 were significantly associated under the allelic model with p values of 0.006 for both SNPs. In addition, haplotype analysis revealed a protective haplotype TACGG to be significantly more frequent in controls (69%) than in PACG cases (59%), with p=0.006.

**Conclusions:**

This study demonstrates an association between *MMP9* SNPs rs3918249 and rs17576 and PACG in the Australian population, suggesting *MMP9* may be involved in the pathogenesis of this blinding disease. Further replication will be helpful in confirming this finding before future clinical translation.

## Introduction

Glaucoma is the second leading cause of blindness worldwide after cataract. It is estimated that 79.6 million people will be affected by 2020, with 11.2 million patients affected with bilateral blindness [1]. Primary angle closure glaucoma (PACG) has high visual morbidity rates and accounts for blindness in about half of all blind glaucoma patients [2].

PACG patients have in common similar anatomic features such as shallow anterior chamber [3], increased lens thickness, anterior position of the lens [4], narrow anterior chamber angles, short axial length [5], and hyperopic refractive error [6]. Wang et al. [7], found that first degree relatives of PACG patients are at 6–9 fold increased risk of developing PACG. The genes responsible for PACG are still unknown.

Recently, the matrix metalloproteinase-9 (*MMP9*) gene was investigated for association with PACG. MMP9 is one of tightly regulated family of zinc dependent enzyme, and is important in remodelling of extra-cellular matrix (ECM) during homeostasis and remodelling [8]. A single study identified an association between a single nucleotide polymorphism (SNP) rs17576 in *MMP9* and PACG in Taiwanese patients [9]. A subsequent study on Singaporean patients did not replicate this association [10], and Cong et al. [11] also failed to find an association between rs17576 and PACG in a Southern Chinese population. However, they reported an association between SNP rs2250889 in *MMP9* and PACG.

In the current study, we aimed to assess tag SNPs in *MMP9* for association with PACG in the Australian Caucasian population.

## Methods

Participants were recruited from Ophthalmology clinics in Australia through the Australian and New Zealand Registry of Advanced Glaucoma (ANZRAG). Approval was obtained from the human research ethics committee of the Southern Adelaide Health Service and Flinders University, and was conducted in accordance with the Declaration of Helsinki and its subsequent revisions. Written informed consent was obtained from each individual. All participants share the same ethnicity (Caucasian).

A total of 107 participants with PACG were recruited. Every participant received a complete eye examination including slit lamp examination of the anterior chamber, gonioscopy, central corneal thickness, visual acuity, measurement of intraocular pressure, fundus examination with special attention to optic disc parameters, and visual field assessment. The diagnosis of PACG was based on the presence of glaucomatous optic neuropathy with cup:disc ratio ≥0.7, peripheral visual loss, presence of at least 180 degrees of closed angle in which the trabecular meshwork is not visible on gonioscopy. Patients with secondary angle closure glaucoma due to uveitis, trauma or lens subluxation were excluded.

The control group, comprising 288 individuals from the Australian population, was recruited from healthy age matched volunteers based in Adelaide, Australia. Controls were all examined and required to have intraocular pressure less than 21 mmHg, normal optic nerve heads with cup:disc ratio of <0.5, normal visual fields and no family history of glaucoma.

Genomic DNA was extracted from 8 ml of venous blood using the QiaAmp Blood Maxi Kit (Qiagen, Valencia, CA). Tag SNPs were selected using the tagger program implemented in Haploview 4.2, to cover the majority of known genetic variation in and around *MMP9* in HapMap using CEU:CEPH (Utah residents with ancestry from northern and western Europe). Tag SNPS were chosen using pairwise tagging, to have an r^2^>0.8 with SNPs displaying a minor allele frequency of 10% in this population. SNPs previously reported to be associated with PACG were force included in the selection of tags. These chosen tag SNPs were: rs3918249 (C/T), rs17576 (G/A), rs3918254 (T/C), rs3787268 (A/G), rs2274756 (A/G). A Bonferroni corrected p value of 0.05/5=0.01 was considered statistically significant.

Genotyping was conducted at the Australian Genome Research Facility using the iPLEX Gold chemistry (Sequenom Inc., San Diego, CA) on an Autoflex mass spectrometer (Sequenom Inc.) at the Australia Genome Research Facility, Brisbane. All analyses were conducted in PLINK [12]. SNPs were assessed for compliance with Hardy–Weinberg equilibrium using the χ^2^ test. Genetic association was assessed under allelic, dominant and recessive models. Where fewer than 5 counts for a given genotype were observed, Fisher’s exact test was used, otherwise a χ^2^ test was used. Haplotypes across the single observed linkage disequilibrium block, as visualized in Haploview using the “confidence interval” block definition [13], were also analyzed for association using PLINK. Multivariate analysis was conducted using logistic regression in PLINK.

## Results

**Figure 1:**
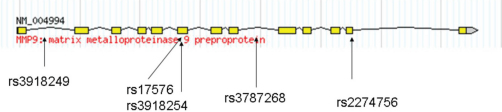
*MMP9* gene schematic representation, indicating tag SNP location. Exons are indicated by boxes and joined by introns indicated by solid lines. Figure adapted from the HapMap website.

Three hundred-ninety five Australian participants, consisting of 107 PACG cases with a mean age of 76±8.2 (females 67% and males 33%), and 288 healthy controls with mean age 69±11.2 (females 53% and males 47%) were genotyped. A total of five SNPs were genotyped at the *MMP9* locus, with the physical location presented in Figure 1. All five SNPs conformed to Hardy–Weinberg equilibrium (p>0.05).

Two *MMP9* SNPs, rs3918249 and rs17576, were significantly associated under an allelic model with p values of 0.006 for both SNPs. SNP rs2274756 showed nominal significance (p=0.017) that did not survive correction for multiple testing (Table 1). The C/C genotype of rs3918249 and the G/G of rs17576 were observed more frequently in PACG patients than in controls (Table 2). Both were associated with marginal significance under dominant and recessive models. Multivariate analyses were conducted controlling for age and gender, the two SNPs remained significantly associated with PACG (p=0.01 for both SNPs).

**Table 1:** Association of *MMP9* tag SNPs with PACG under an allelic model. Position on chromosome 20 is given in base pairs along with frequency of Allele 1 in cases and controls.

**SNP**	**Position (bp)**	**Allele 1/2**	**Case**	**Control**	**p-value**	**OR (95% CI)**
rs3918249	Chr20:44071543	C/T	0.411	0.305	**0.006**	1.5 (1.1–2.2)
rs17576	Chr20:44073632	G/A	0.408	0.304	**0.006**	1.5 (1.1–2.2)
rs3918254	Chr20:44073798	T/C	0.004	0.001	0.469	2.6 (0.1–43)
rs3787268	Chr20:44075138	A/G	0.231	0.192	0.232	1.2 (0.8–1.8)
rs2274756	Chr20:44076518	A/G	0.174	0.111	0.017	1.7 (1.1–2.6)

A p<0.01 in bold values considered to be statistically significant. bp=base pair, OR=odds ratio, 95% CI=95% confidence interval.

**Table 2:** Genotype frequencies of *MMP9* SNPs, and association under dominant and recessive models.

**SNP**	**Genotype**	**Cases n (%)**	**Controls n (%)**	**p-value dominant**	**p-value recessive**
rs3918249	CC	19 (17%)	27 (10%)	0.02	0.04
	CT	49 (46%)	109 (41%)		
	TT	38 (36%)	131 (49%)		
rs17576	GG	18 (17%)	27 (10%)	0.02	0.05
	GA	49 (47%)	109 (41%)		
	AA	37 (35%)	132 (49%)		
rs3918254*	TT	0	0	0.47	1
	TC	1 (0.9%)	1 (0.3%)		
	CC	105 (99%)	282 (99%)		
rs3787268*	AA	7 (6%)	9 (3%)	0.41	0.16
	AG	35 (33%)	85 (32%)		
	GG	64 (60%)	174 (65%)		
rs2274756	AA	4 (4%)	4 (1%)	0.04	0.23
	AG	29 (27%)	51 (19%)		
	GG	73 (69%)	213 (79%)		

*indicates Fisher’s exact test was used.

**Figure 2:**
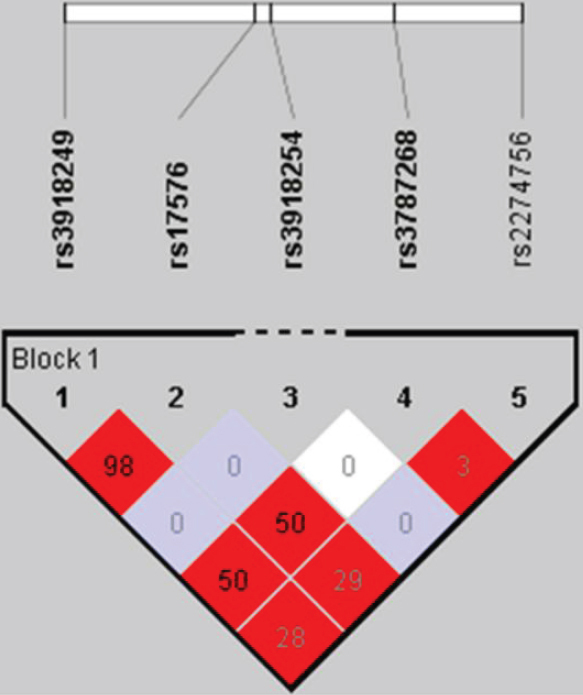
The haplotype block structure of the tag SNPs of *MMP9*. The number in the box represents the r^2^ value. Values in light blue boxes do not reach statistical significance.

Analysis of the linkage disequilibrium structure between the five tag SNPs showed one haplotype block (Figure 2). Haplotype analysis revealed three common haplotypes in this population. The frequency of TACGG was significantly higher in controls (69%) than in PACG cases (59%), p=0.006, and remained significant after Bonferroni correction for the three haplotypes observed (p=0.018). The CGCGA haplotype was more frequent in PACG cases than controls (17% versus 11% respectively, p=0.035), but did not remain significant following multiple testing correction (corrected p=0.105) (Table 3).

**Table 3:** Haplotype frequencies in PACG patients and controls.

**Haplotype**	**Cases**	**Controls**	**OR (95% CI)**	**p-value**
C G C G A	0.17	0.11	1.62 (1–2.5)	0.035
T A C G G	0.59	0.69	0.63 (0.5–0.9)	0.006
C G C A G	0.23	0.19	1.26 (0.8–1.8)	0.239

95% CI=95% confidence interval. A p<0.05 level is considered statistically significant.

## Discussion

*MMP9* is located on chromosome 20q11.2-q13.1 and contains 13 exons. MMP9 protein plays an important role in extracellular matrix remodelling by cleaving denatured collagen and type IV collagen in the basement membrane. Regulation of *MMP9* occurs at the transcriptional level, secondary to pro-inflammatory cytokines [14], while the activation and inhibition of the secreted pro-enzyme (ProMMP-9), controls the post-transcriptional regulation [15].

SNP rs17576 is located in exon 6 of *MMP9*, where the mutation leads to the substitution of positively charged amino-acid (arginine) by an uncharged amino acid (glutamine) at position 279 [16]. This non-synonymous substitution is situated in the coding sequence of a highly conserved gelatinase-specific fibronectin type II domain (FN2) [17]. The FN2 is one of three types of the internal repeats that combine to form larger domains within fibronectin (a plasma protein that binds various cell surface compounds such as collagen, fibrin, heparin, DNA and actin). This domain in MMP9 is responsible for the collagen affinity of MMP9 [18]. The precise impact of this polymorphism on protein function is currently unknown, but it has been suggested that it could lead to partial loss of function in ECM remodelling which occurs during eye growth and development [9].

Our study found a significant association of two SNPs in *MMP9* with PACG in the Australian Caucasian population; rs17576 (G/A; p=0.006) and rs3918249 (C/T; p=0.006), these SNPs remained statistically significant independent of age and sex. These two SNPs are in strong linkage disequilibrium (r^2^=0.98). The minor allele of each SNP (G and C, respectively) is associated with PACG under the allelic and dominant models. These risk alleles are split across two common haplotypes, only one of which is associated with PACG. The most significantly associated haplotype contains the common allele at both SNPs and appears to be protective with a haplotype frequency of 69% in controls and only 59% in cases, p=0.006.

Previous studies of the association of SNPs in *MMP9* with PACG in different populations have been reported. Wang et al. [9], found an association between rs17576 and acute PACG in Taiwanese populations. They postulated that the gene activity may have been down regulated in PACG patients, leading to reduction of MMP9 activity in ECM remodelling during ocular development and thus shorter axial length. However, other studies on Singaporean and Southern Chinese patients failed to replicate this finding [10,11]. Cong et al. [11], also identified an association of *MMP9* SNP rs2550889 with PACG in a Southern Chinese population. This SNP was not included in our study, as it was unable to be genotyped in a multiplex with the other SNPs. However, it is in strong linkage disequilibrium with our two associated tag SNPs (rs17576 and rs3918249, r^2^=0.85 and 0.86, respectively, in the HapMap, CEU sample), suggesting a similar finding in the current study. Further replications are required to directly examine the association of rs2550889 SNP with PACG in Australian individuals.

Interestingly, these three previous studies all in Asian populations showed that the A/A genotype of SNP rs17576 was more common in PACG cases than in normal controls. Thus, the minor A allele conferred risk for PACG. In the current Caucasian study, we find that the A allele confers protection against PACG and is the commoner allele in this population. There is a well documented difference in allele frequency across populations at this SNP. Asian populations range from 0.22 to 0.30 [9-11,19,20], while the frequency in Caucasians is approximately 0.65 [21-24]. This is also observed in HapMap.

The opposite association of an allele or genotype of the same SNP with disease could be due to different functional effects among different ethnic groups, or the heterogeneous effect of the same variant such as genetic background or environmental factors [25]. This “flip-flop” association may indicate that rs17576 is not the causative allele despite being a non-synonymous change (Gln279Arg), but that the risk variants occur on different genetic backgrounds in different ethnicities. Additionally, the fact that this variant is predicted to be benign or tolerated by both PolyPhen and SIFT supports this hypothesis. Examples of such “flip-flop” associations have been previously reported [25], and are well established in ophthalmology for the coding lysyl oxidase-like 1 (*LOXL1*) variant rs1048661 (R141L) SNP associated with pseudo-exfoliation syndrome in opposite directions in Japanese population compared with Caucasians [26]. Alternatively, the findings could represent type 1 errors that do not replicate in further studies.

In Conclusion, our results show a significant association between *MMP9* polymorphisms and PACG in the Australian Caucasian population, although the mechanism of *MMP9* in causing this blinding disease is undetermined. This is the first study to suggest an association between *MMP9* polymorphisms and PACG in the Australian Caucasian population, and one of the first studies to investigate angle closure glaucoma genetics in the Caucasian population. Additional replication studies in populations of similar ethnicity to the Australian Caucasian population are necessary to confirm this association.
